# Influence of Cyclic Loading on the Removal Torque of Unique Subperiosteal Implant Screws

**DOI:** 10.3390/jfb16090306

**Published:** 2025-08-22

**Authors:** Ádám Vörös, Klaudia Kulcsár, Dávid Pammer, Ibolya Zsoldos

**Affiliations:** 1 Department of Materials Science and Technology, Széchenyi István University, 9026 Győr, Hungary; 2 Alba Regia Technical Faculty, Óbuda University, 8000 Székesfehérvár, Hungary; kulcsar.klaudia@amk.uni-obuda.hu; 3 PaB Kereskedelmi és Fejlesztő Kft., 1116 Budapest, Hungary; david.pammer@pab.hu

**Keywords:** subperiosteal implant, implantology, screw loosening, screw tightening

## Abstract

During the investigation, the effect of screw tightening torque on the potential loosening of screws under load was examined in the case of custom-made subperiosteal implants. The study focused on the connection screws between the implant components, testing the commonly applied tightening torques of 15 Ncm and 30 Ncm. Mastication was simulated using a custom-designed, PLC-controlled testing device, which allowed for the reproduction of variable numbers, forces, and speeds of bite cycles. With this device, six different scenarios were tested, including 500, 2000, and 10,000 bite cycles, under both constant and variable bite forces. A caliper was used to record potential length changes of the screws, force sensors measured the bite forces, and calibrated torque screwdrivers were used to verify the loosening torques. Based on the analysis of the measured data, it was concluded that for the M1.8 screws tested, a tightening torque of 15 Ncm does not provide sufficient resistance against loosening, whereas 30 Ncm offers adequate stability.

## 1. Introduction

Dental implants are used for the treatment of partially and completely edentulous patients, and they demonstrate high success rates in both the medium and long term [1,2,3,4].

Titanium (Ti)-based dental implants are used for the functional and aesthetic replacement of missing teeth without compromising adjacent teeth. Prosthetic restorations supported by implants provide natural appearance and stability, making them widely used in modern dentistry [5]. From a biomechanical perspective, however, the load-transfer characteristics of osseointegrated implants differ from those of natural teeth. Since the implant is anchored directly into the cortical and cancellous bone, the stress-buffering function of the periodontal ligament is absent. As a result, occlusal forces are transmitted directly to the surrounding bone tissue [6].

This direct force transmission may, over time, lead to increased bone loading, bone resorption, and the formation of peri-implant bone defects. The implant–abutment interface plays a critical role, as it significantly affects the long-term stability of the entire system [7]. Mechanical loading—especially repetitive occlusal forces—can induce micromovements, deformations, microcracks, and even screw loosening or fracture [8]. These factors may negatively impact the longevity and clinical success of the implant, underscoring the importance of accurately understanding and optimizing its biomechanical behavior for successful implant-based rehabilitation [7].

The screws used for securing the implant and the abutment must be tightened with a torque value specified in Ncm, as recommended by the manufacturer [9]. For the initial step of screw tightening, a so-called preload torque is applied. This ensures improved adaptation at the connection interfaces [10]. Inadequate fit at these interfaces can lead to the development of high stress concentrations, which may result in screw loosening [11]. Screw loosening remains one of the most common complications associated with dental implants to this day [12,13]. A potential screw loosening causes displacement between the osseointegrated implant wire and the interface. Such complications may result in the loss of the prosthetic restoration supported by the implant. Furthermore, there is a potential risk of damage to the implant itself, the abutment, or the surrounding soft and osseous tissues. Studies have shown that a higher preload requires greater force to loosen the screws [14].

In the study by Körtvélyessy et al., the effect of the taper angle in implant–abutment connections was investigated with regard to the mechanical stability and clinical performance of dental implants. The analysis focused on how different taper angles influence the long-term durability and clinical success of dental implants. Two primary issues were addressed: the loosening of the retaining screw observed during mechanical testing and horizontal deformation detected via finite element analysis. The results indicated that an increase in the taper angle led to a reduction in removal torque, thereby decreasing the extent of loosening under load. Smaller taper angles resulted in greater torque loss and deformation, whereas larger angles were associated with more stable connections and reduced compression rates. It can be concluded that optimizing the taper angle plays a key role in enhancing prosthetic stability and preventing screw loosening [15].

According to the study by Rico-Coderch et al., implant–abutment connections may be designed with different taper angles, which can influence their mechanical stability. The aim of the research was to investigate the mechanical behavior of implant–abutment interfaces, with particular focus on screw loosening in relation to varying taper angles. Five types of implant samples with different taper angles (24°, 35°, 55°, 75°, and 90°) were tested under both static and dynamic loading conditions, with a total of thirty-five samples. All retaining screws were uniformly tightened to a torque of 35 Ncm. During static testing, a 500 N load was applied over 20 s, while dynamic testing involved 15,000 cycles with a load of 250 ± 150 N. The results showed a consistent trend: greater taper angles were associated with reduced screw loosening. It was concluded that increasing the taper angle has a beneficial effect on connection stability, which may contribute to the long-term, secure function of prosthetic restorations [16].

### 1.1. Endosteal Dental Implants

Screw-type implants (Endostel Dental Implants) require an adequate amount of bone volume in the patient to ensure proper placement [17].

Endosseous (or endosteal) dental implants, surgically anchored into the alveolar bone, represent the dominant solution in current implantology. They are employed both for single-tooth replacement and for supporting multi-unit prosthetic restorations. Owing to advances in biomimetic design, the morphology of these implants closely replicates that of natural tooth roots, promoting effective integration with the surrounding bone tissue. Among various types, screw-shaped implants are the most utilized in clinical practice.

From a structural standpoint, implants can be categorized into one-piece and two-piece systems. In the one-piece configuration, the implant body and the prosthetic abutment are manufactured as a single unit. These are typically indicated for the anterior region and are used exclusively for single crowns. In contrast, two-piece implants consist of a root-form fixture placed into the bone and a separate abutment that supports the prosthesis.

The surgical procedure generally involves two stages. First, the implant fixture is inserted into the bone and sealed with a cover screw. Following soft tissue closure, a healing period of several months is required for osseointegration. In the second stage, the cover screw is removed and replaced by a healing abutment; once soft tissue has adapted, the definitive abutment is secured into place.

Two-piece implants offer improved biomechanical stability and allow for the use of angled abutments, increasing prosthetic versatility. A key clinical advantage is their modularity: if the prosthetic component becomes damaged, it can be replaced without removing the osseointegrated fixture. In contrast, failure or damage of one-piece implants typically necessitates complete removal and surgical reintervention.

### 1.2. Subperiosteal Implants

The concept of subperiosteal implantology was first developed by Gustav Dahl in Sweden in the early 1940s [18]. However, the technique did not gain widespread acceptance due to the complexity of the surgical procedure and the inadequate adaptation of the implant to the underlying bone, which led to unfavorable long-term clinical outcomes [19]. The use of this method further declined with the growing prevalence of endosseous implantation techniques [20].

Subperiosteal implants offer a valuable alternative in oral rehabilitation, particularly in cases where patients present with insufficient bone volume for conventional implant placement. These custom-designed implants aim to restore masticatory function in individuals with multiple adjacent missing teeth and inadequate bone quality or quantity that precludes the use of endosseous screw-type implants.

Subperiosteal frameworks can be utilized in both partial and complete edentulism of the maxilla or mandible. Their design is based on high-resolution CBCT imaging, and fabrication is achieved through computer-aided design and manufacturing (CAD/CAM) workflows. The implant structure, typically composed of a Ti-6Al-4V titanium alloy, conforms precisely to the surface of the alveolar bone. The material ensures sufficient mechanical strength and biocompatibility, while also facilitating osseointegration.

Advantages of subperiosteal implants include:

- Simultaneous bone augmentation, implant placement, and prosthetic restoration in a single procedure.
- Sufficient bone volume is not necessary, as they can be placed directly onto the bone surface.
- Significantly reduced healing time.
- Precise adaptation to the existing bone surface.
- Lower risk of surgical complications.
- Reduced postoperative pain and morbidity.
- Possibility of immediate provisional prosthetic loading [21,22].

Numerous studies have investigated screw loosening in dental implants, the causes of microgap formation, and the biological implications of these phenomena [23,24,25,26].

In the study by De Riu et al., a custom-designed subperiosteal implant anchored to the temporalis muscle flap was used in a patient for whom free flap reconstruction was not feasible. The procedure enabled the restoration of separation between the oral and nasal cavities, provided a stable foundation for prosthetic rehabilitation, and also offered adequate soft tissue support in the midfacial region. Subperiosteal implants may represent a safe and effective alternative for primary maxillary reconstruction in patients who are not candidates for free flap bone grafting. In these complex cases, the technique effectively supports the soft tissues of the midface while providing stable anchorage for dental prostheses or obturators, thereby contributing significantly to the patient’s functional rehabilitation and quality of life [27].

The aim of this study was to determine the optimal torque value required to securely tighten the screws in order to minimize the risk of loosening under mechanical loading, while simultaneously avoiding screw failure (such as head fracture or internal hex deformation).

## 2. Materials and Methods

### 2.1. Description of the Issue

During a previous investigation, we observed that several screws used to connect the abutments to the implant interfaces began to loosen following fatigue testing of the subperiosteal implant. Although the primary finding was that some screw dimensions deviated from the expected values (e.g., internal hex depth, screw shank diameter), this prompted us to conduct a more thorough investigation into the issue of screw loosening.

**Figure 1:**
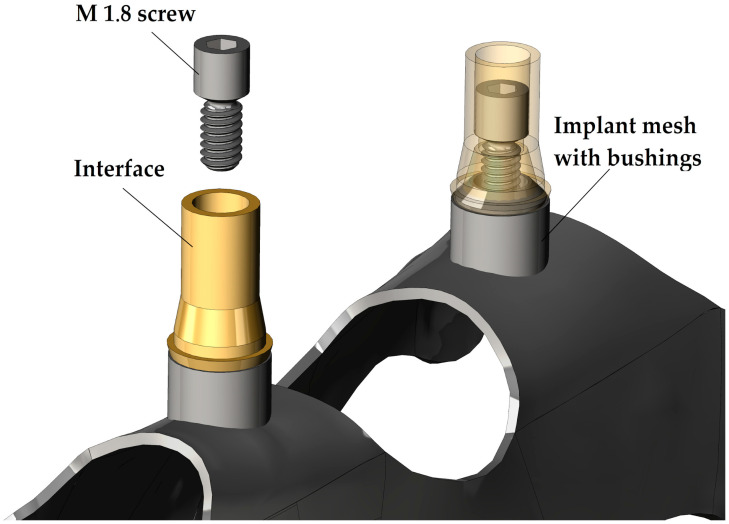
Structure of the subperiosteal implants and position of the investigated screw.

The structure of a subperiosteal implant and the position of the investigated screws in the case of a dental implant are visible in Figure 1. As shown in the image, these fixation screws do not come into direct contact with the bone tissue (nor with soft tissue); therefore, osseointegration does not occur. All the screws were manufactured by turning, based on the actual screw design provided by the implant manufacturer.

In general, for dental implant systems, the recommended tightening torque for retention screws in the M1.8, M2, and M2.5 size range typically falls between 15 and 30 Ncm (±5 Ncm). Each manufacturer defines a specific torque value for their screws based on their proprietary design, material selection, functional requirements, and technical know-how.

Some researchers have reported that even at high tightening torque values, microgaps may still form between the connecting surfaces, potentially leading to mechanical failure [21,22,23,24,25,26,27,28,29,30].

These microgaps provide a favorable environment for microorganisms, creating a slippery interface due to microbiological activity, which may in turn lead to infections [4].

**Figure 2:**
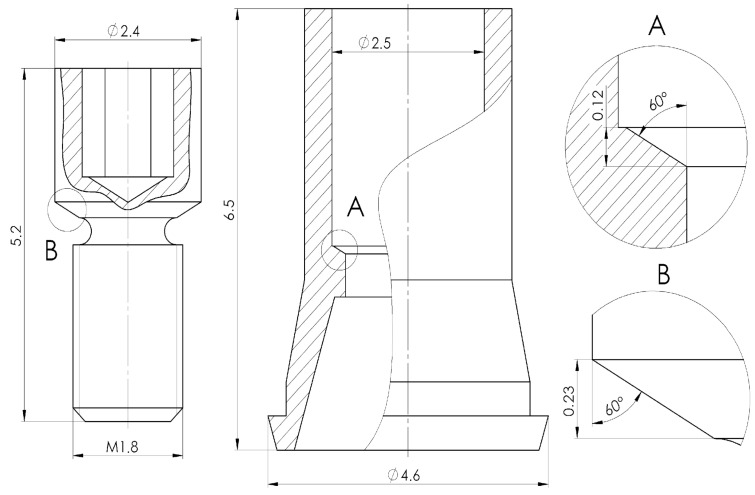
Design of connection between interface and screw.

To achieve the best bacterial leakage prevention and avoid screw loosening, manufacturers use various conical joint geometries between the screw and the interface. Some use self-locking conical connections, where the half-cone angle ranges from 2° to 5°. This method effectively prevents screw loosening; however, if joint separation is required during medical surgery, the surgeon will need a special tool and more time to perform the procedure. In Figure 2, we can see the key dimensions of the screw and interface design. The dimensions of joining surfaces are visible in the right of the figure in detailed views. The interface-to-screw connection surface features a conical geometry with the cone dimensions illustrated in details A and B of Figure 2. Both components were produced by turning with a standard surface roughness of Ra 1.6. No post-processing was applied to the screws. In contrast, the interfaces were anodized after turning, resulting in a yellow, slightly glossy surface. This coloration indicates the formation of a titanium oxide layer with an estimated thickness of 40–50 nanometers.

During the chewing process, the subperiosteal implant is primarily subjected to compressive and flexural stress. The direction of these loads is constantly changing and is not always aligned with the longitudinal axis of the screw and the interface. If the connection between the screws, interfaces, and bushings is inadequate, these dynamic loads may lead to screw loosening.

The possible reasons behind inappropriate connections:

- Wrong screw design
- Wrong interface design
- Manufacturing failure
- Raw material failure
- Inappropriate screw tightening value

### 2.2. Overview of the Test Equipment

**Figure 3:**
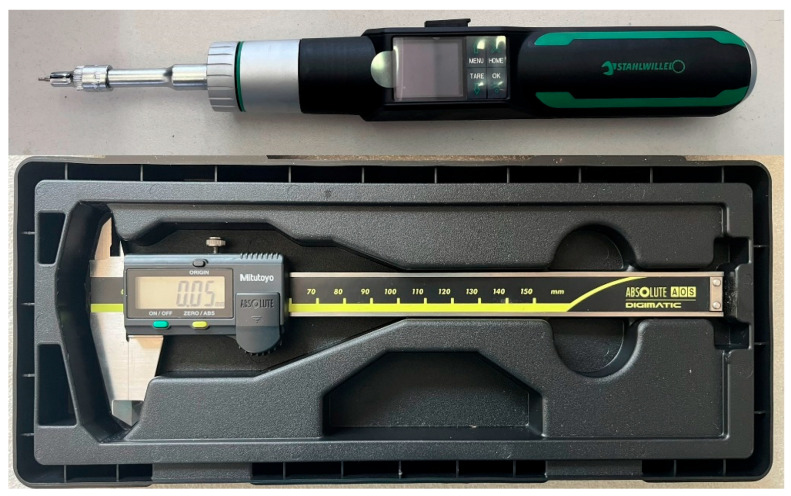
Measuring tools.

During the tests, several types of measuring instruments and devices were used. The manual measuring tools that were used for length, diameter, and tightening/loosening torque measuring are visible in Figure 3.

The Stahlwille Torsiotronic 1.2 torque wrench has several programs. The wrench is capable of tightening screws with a defined torque between 12 and 120 Ncm. The torque screwdriver is calibrated for measurements in the range of 12 to 120 Ncm. It can transmit values below this range as well, but its uncertainty in that region is unknown. It is also possible to measure the maximum loosening torque during screw loosening.

**Figure 4:**
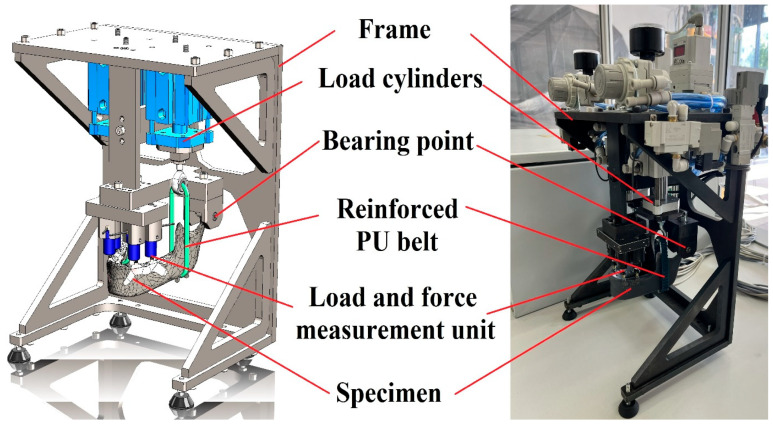
Main parts of the test bench.

All the other measuring devices are part of the individual testing device that we designed and built. The main components of the test bench are shown in Figure 4 [31].

This test device enables us to do mechanical tests on the dental implant. Compressive loading can be applied at a minimum of one and a maximum of four points. Typically, the upper surfaces of the interface components are used as loading points, where the forces generated during biting can be measured.

The device is operated via PLC control, allowing the user to create custom testing protocols that simulate mastication through multiple bite cycles.

In the recipe editing menu, the operator could define the following parameters with distinct values assigned for each cycle:

- Number of bites in the cycle [pcs].
- Loading force of left cylinder [N].
- Loading force of right cylinder [N].
- Long of the bite [s].
- Time between two bites (relaxation) [s].
- Sampling frequency [sample/“x” bites].

Table 1 summarizes the measured dimensions and the used devices.

### 2.3. Overview of the Test Process and Specimen

The test process itself consisted of five individual steps. The first step was the assembly of the test specimen. During the initial assembly, it was important that the screws were only lightly tightened, and the final tightening torque was only applied using a torque screwdriver.

The specimen consisted of a 3D-printed photopolymer mandible, two subperiosteal implants (adhered to the mandible with two-component pattex repair universal epoxy glue; the adhesive’s tensile strength after curing was approximately 130 kg/cm^2^ [~13 MPa] at 24 °C), and four interfaces that were fixed with the tested screws.

The 3D-printed mandible was fabricated from a virtual, toothless, anatomical 3D CAD model, which was designed to replicate the mandible surface and structure (e.g., force arms and attachment points) of a real mandible. SLA 3D printing technology has the capacity to produce mandible models with adequate detail and material quality for testing purposes [32].

**Figure 5:**
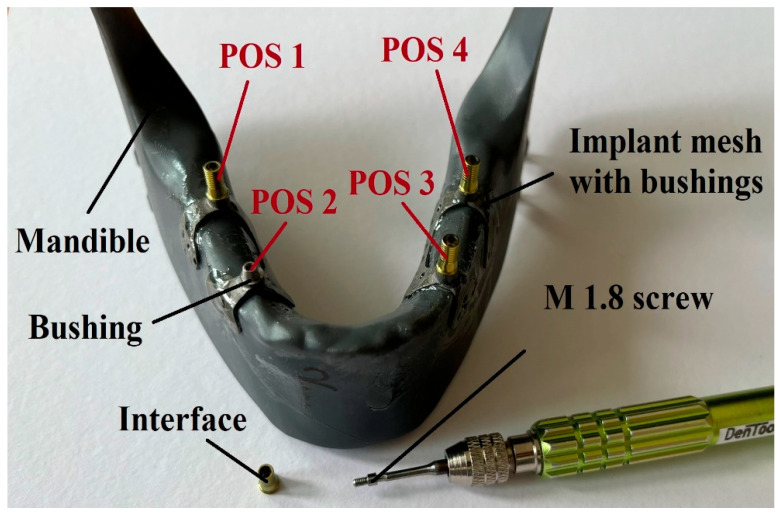
Specimen during preparation.

3D printing was performed with a layer thickness of 50 µm and Tough 2000 material. The mandible had a completely solid, unstructured cross-section, and the support structure connection points for 3D printing were located on surfaces that were irrelevant for the test. Following the curing process in accordance with the manufacturer’s guidelines, the material demonstrated adequate strength properties, enabling the mandible model to withstand the test without failure [33,34]. A specimen with these elements is shown in Figure 5.

Similarly, the designated test points of the test specimen are shown in Figure 5. The names displayed in the figure were used during the tests. The naming of the test points was always based on the orientation as if the implant occupied its position after installation:

- POS 1: Right posterior
- POS 2: Right anterior
- POS 3: Left anterior
- POS 4: Left posterior

When the assembly of the specimen was ready, then the next step was the mounting of the specimen onto the test bench. After this, the test recipe needed to be selected or created. The next step was the adjustment of the desired torque value on the torque screwdriver and the tightening of the screws to the specified torque. Once all of this was done, the test program simulating mastication could be started. After that, only three steps remained: the first was measuring the screw loosening torque using a torque screwdriver, the second was disassembling the parts completely, and finally, the measuring of any length changes in the screws using a caliper. 

**Figure 6:**
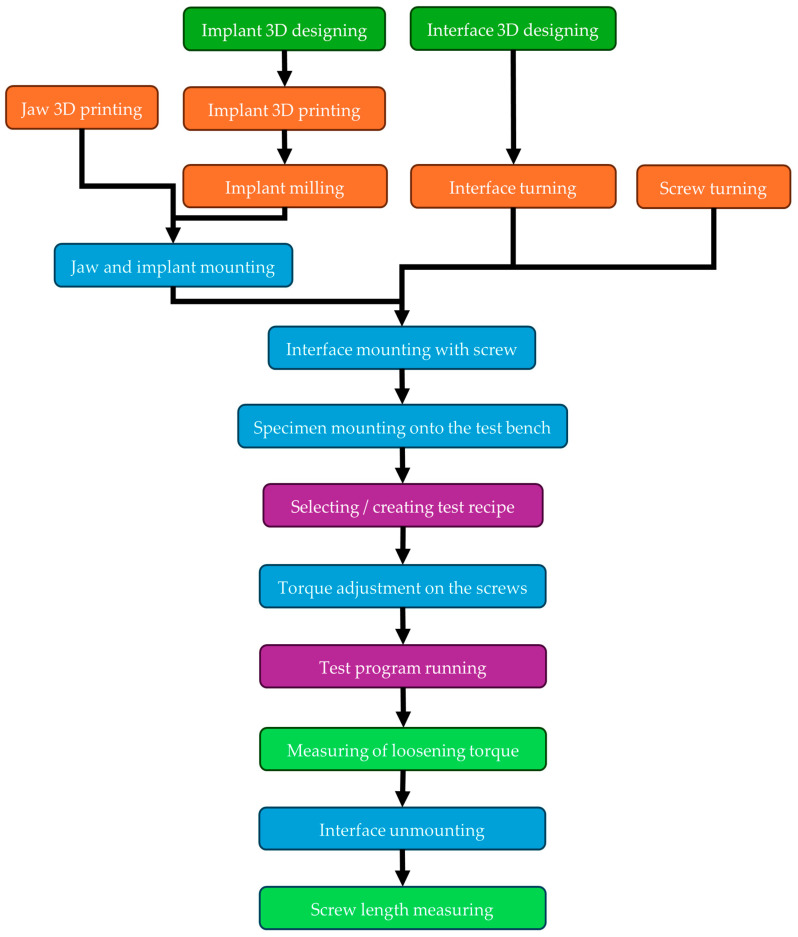
Test process flowchart.

The whole process flowchart (from specimen design to measured value) can be seen in Figure 6, where the color representation is as follows:

- Dark green: Design
- Orange: Manufacturing
- Blue: Assembling
- Purple: Testing
- Light green: Measuring

During the study, two different tightening torque values were examined: the commonly used upper and lower limits:

- 15 Ncm (±1 Ncm)
- 30 Ncm (±1.5 Ncm)

A tightening was considered successful if it fell within the tolerance range shown above.

### 2.4. Overview of Used Test Programs

As outlined in Section 2.1 of the test procedures, several parameters could be adjusted. The testing involved five different programs, each with a distinct number of bites and a different fatigue method (constant or variable). In the case of the variable method, five different cycles were used with varying biting parameters (force, bite length, and relaxation length). In contrast, for the constant method, all bites were performed using the same parameters throughout the test.

Each test recipe consists of either one or five cycles. The parameters for the 500-bite constant test are shown in Table 2, and those for the 500-bite variable test in Table 3. The parameters for the 2000-bite constant test are listed in Table 4, and those for the 2000-bite variable test in Table 5. Table 6 presents the parameters for the 10,000-bite variable test.

It is important to mention that during the research, all examinations (tightening, testing, loosening) were repeated three times, except for the 10,000-bite variable tests and the short tests without fatigue testing.

The reason is the runtime requirements of the individual tests, which imposed limitations, as the testing device requires continuous engineering supervision during operation. Since the testing equipment is still in the prototype stage, it cannot be operated unattended due to safety considerations.

Approximate runtime of individual tests:

- 500 bites: ~20 min
- 2000 bites: ~1 h 20 min
- 10,000 bites: ~6 h 40 min

Table 7 shows how many times each type of test was repeated. It also indicates how many data points were generated during the research and how many of them were suitable for further analysis.

## 3. Discussion

### 3.1. Loosening Tests Without Fatigue Testing

In order to determine the relationship between the screw tightening torques and the loosening torques required to loosen screw connections, eight different screws on a single specimen were tightened 10 times each while monitoring any possible changes in screw length, as well as the loosening torque requirement. Out of the eight screws, a target tightening torque of 15 Ncm was applied to four screws and 30 Ncm to the other four.

It was observed during these 10 cycles that the total length of the screws (5.2 mm) did not change. Therefore, it can be concluded that the screws do not undergo any permanent deformation during the 10 tightening cycles. The length measuring method is shown in Figure 3.

At the same time, it was also observed that although the number of tightenings did not affect the loosening torque, the position of the screws on the specimen (POS1–POS4, Figure 5) significantly influenced the expected loosening torque values. The reason behind this is the variation in manufacturing dimensions within the specified tolerances. It was also observed that the screw in the left anterior position produced an outlier result; in this single case, the required loosening torque exceeded the applied tightening torque. This anomaly is because the screw hole was not perfectly concentric with the mating conical surface. Due to this inaccuracy, this measurement point will be excluded from further analysis.

The expected values and minimum loosening torque can be seen in Table 8.

Based on the findings, it was calculated that the mean loosening torque is 11.2% lower than the tightening torque in the case of 15 Ncm tightening and 9.0% lower in the case of 30 Ncm tightening. The deviation between these two values is small considering that the connection between the two elements (screw and insert) is different in the case of each tightening. In this case, small screw sizes, dust or burr particles can be a significant measurable deviation between the measured values. The human factor during measurement cannot be neglected either. Unfortunately, in these cases, there is no possibility to repeat the measurements, as a screw can only be loosened once. Another possible cause of further inaccuracy could be that measurement uncertainty under 15 Ncm can be bigger than in the case of the measuring range of 12–120 Ncm (display deviation value ±4%).

### 3.2. Loosening Tests with 15 Ncm and 30 Ncm Tightening Torque

In the screw loosening tests, it is important to emphasize that the screws under investigation establish a connection between a titanium implant and a titanium abutment. Therefore, osseointegration does not occur between the contacting surfaces of the components, making the evaluation of screw loosening more justified.

**Figure 7:**
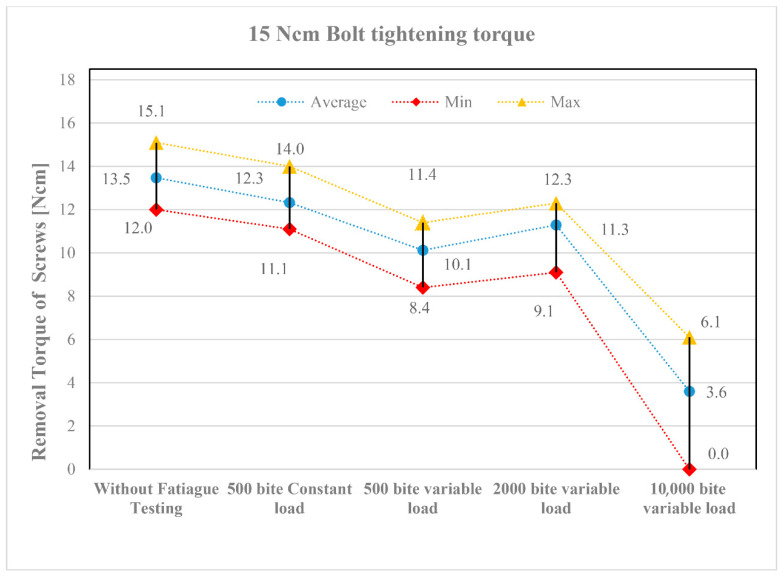
Loosening torque values of screws tightened to 15 Ncm.

**Figure 8:**
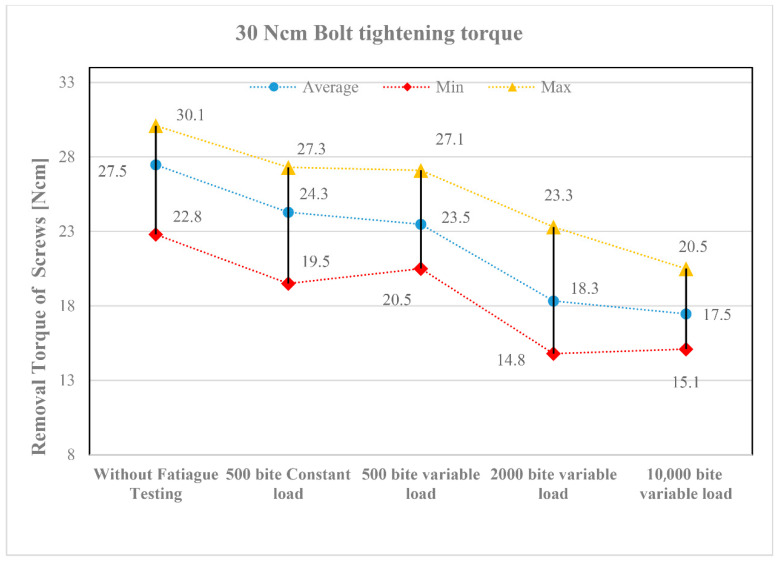
Loosening torque values of screws tightened to 30 Ncm.

The results of the screw loosening tests are summarized in diagrams shown in Figure 7 and Figure 8. Figure 7 presents the results for a tightening torque of 15 Ncm, while Figure 8 shows those for 30 Ncm. The horizontal axis represents the different types of loading tests in the order listed in Table 7. The vertical axis indicates the torque values measured after loading (loosening torque). Table 7 also shows how many repetitions (1, 3, or 10) were performed for each test type. In the diagrams, the lowest torque values are marked with red, the highest with yellow, and the average values with blue.

Despite the three repetitions, the torque values show a noticeable scatter. This primarily resulted from the screws being placed in different implant positions (see the layout in Figure 5). Although the applied loading was identical, slight differences in function still occurred. Nevertheless, the following trends can be observed in the diagrams:

- All fatigue testing resulted in a smaller or greater degree of screw loosening compared to the case without fatigue testing (“Without Fatigue Testing”), meaning that the torque values measured after the tests were always lower than those without fatigue loading.
- It should be emphasized, however, that when the magnitude of the applied loading varied during fatigue testing (“500 bite variable load”), a more significant loosening was already evident at the lower tightening torque of 15 Ncm, indicating a greater reduction in the measured torque values.
- When the number of loading cycles increased to 2000 (“2000 bite variable load”), loosening remained minor primarily at the higher tightening torque (30 Ncm). However, when the number of cycles was further increased to 10,000, clear differences emerged between the lower (15 Ncm) and higher (30 Ncm) tightening torques. At 15 Ncm, a strong or complete screw loosening was observed, whereas at 30 Ncm, the loosening torque stabilized, indicating that the screws can be considered reliable in the long term.

The tightening torque of the screws is of crucial importance in the assembly of dental implants. Due to masticatory function, the components are exposed to repeated loading, which may lead to screw loosening over time. The results of the fatigue tests designed to simulate these repeated loads demonstrated that a low tightening torque (15 Ncm) may result in severe or complete screw loosening. In contrast, a tightening torque of 30 Ncm can lead to a stable torque value, allowing for predictable long-term performance of the implant. For dental subperiosteal implants, particular attention should be paid to the applied tightening torque when connecting the implant and abutment using screws.

For the 30 Ncm test, the same specimen was used but with new screws. The entire process was the same as in the tests with 15 Ncm tightening. The results are shown in Figure 8.

## 4. Conclusions

The results of the fatigue tightening and loosening tests conducted during the study suggest that the selection of an appropriate tightening torque plays a critical role in influencing the expected loosening torque. Moreover, the findings indicate that during fatigue loading, the type of loading—whether constant (i.e., uniform force applied at regular intervals) or variable (i.e., varying force and irregular timing)—has a relatively minor impact on the loosening torque requirement. In contrast, the number of loading cycles appears to be the predominant factor affecting screw stability.

In light of the study results, the research group recommends using a tightening torque of 30 Ncm for the M1.8 fixing screws to minimize the likelihood of loosening after implantation. This recommendation is based on the observation that the measured loosening torque remained stable and nearly identical after both the 2000- and 10,000-cycle bite tests.

In the present study, the authors found that, as expected, increasing the tightening torque reduces the likelihood of screw loosening and the potential loss of the screw and/or implant. However, care must be taken to avoid the use of excessively high tightening torque values, as these may result in damage to the external threads of the screw and/or the internal threads of the implant, and in some cases, fracture of the screw head.

It was also noted that the geometry of the internal drive recess within the screw head plays a decisive role in determining the maximum allowable tightening torque. Inappropriate matching of the recess dimensions and the driver geometry can lead to localized stress concentrations during tightening, which in turn may cause premature wear or failure. Therefore, the selection of the tightening torque should not only consider the implant–abutment interface and clinical loading conditions but also the mechanical limits defined by the screw head design.

These results are particularly relevant for patients with strong bite forces and for prosthetic treatments involving custom-made subperiosteal implants.

## Figures and Tables

**Table 1:** Used measuring devices.

Dimension	Name	Unit of Measurement	Type
Length	Caliper	[mm]	Mitutoyo CD-15APX (Mitutoyo Shop, Budapest, Hungary)
Diameter	Caliper	[mm]	Mitutoyo CD-15APX
Awaken force	Load cell	[N]	Kaliber 6021 100 kg (Kaliber Kft., Budapest, Hungary)
Cylinder stroke	Position sensor	[mm]	SMC D-MP025B (Toolimpex, Budapest, Hungary)
Tightening/loosening torque	torque screwdriver	[Ncm]	Stahlwille Torsiotronic 1.2 (SMC Europe, Törökbálint, Hungary)

**Table 2:** 500-bite constant test recipe.

Cycle	Number of Bites [pcs]	Right and Left Cylinder Force [N]	Bite Length [s]	Relaxation Length [s]
1	500	500	1	0

**Table 3:** 500-bite variable test recipe.

Cycle	Number of Bites [pcs]	Right and Left Cylinder Force [N]	Bite Length [s]	Relaxation Length [s]
1	45	300	1	1
2	10	650	10	2
3	300	300	1	0
4	45	750	5	1
5	100	300	1	0

**Table 4:** 2000-bite constant test recipe.

Cycle	Number of Bites [pcs]	Right and Left Cylinder Force [N]	Bite Length [s]	Relaxation Length [s]
1	2000	500	1	0

**Table 5:** 2000-bite variable test recipe.

Cycle	Number of Bites [pcs]	Right and Left Cylinder Force [N]	Bite Length [s]	Relaxation Length [s]
1	180	300	1	1
2	40	650	10	2
3	1200	300	1	0
4	180	750	5	1
5	400	300	1	0

**Table 6:** 10,000-bite variable test recipe.

Cycle	Number of Bites [pcs]	Right and Left Cylinder Force [N]	Bite Length [s]	Relaxation Length [s]
1	900	300	1	1
2	200	650	10	2
3	6000	300	1	0
4	900	750	5	1
5	2000	300	1	0

**Table 7:** Measured values during the research.

Test Type	Repetitions	Data Type	Data Suitable for Analysis
Without fatigue testing, 15 Ncm	10 times	Loosening torque	40
		Screw length	16
Without fatigue testing, 30 Ncm	10 times	Loosening torque	40
		Screw length	16
500 bites constant, 15 Ncm	3 times	Reaction force	150
		Loosening torque	8
		Screw length	12
500 bites constant, 30 Ncm	3 times	Reaction force	150
		Loosening torque	8
		Screw length	12
500 bites variable, 15 Ncm	3 times	Reaction force	99
		Loosening torque	8
		Screw length	12
500 bites variable, 30 Ncm	3 times	Reaction force	99
		Loosening torque	8
		Screw length	12
2000 bites variable, 15 Ncm	3 times	Reaction force	612
		Loosening torque	8
		Screw length	12
2000 bites variable, 30 Ncm	3 times	Reaction force	612
		Loosening torque	8
		Screw length	12
10,000 bites variable, 15 Ncm	1 time	Reaction force	3060
		Loosening torque	3
		Screw length	6
10,000 bites variable, 30 Ncm	1 time	Reaction force	3060
		Loosening torque	3
		Screw length	6
Sum	40	-	8092
Loosening torque data count	-	-	134

**Table 8:** Values of loosening torque without fatigue testing.

Screw Position	15 Ncm Tightening Torque		30 Ncm Tightening Torque	
	Mean Value [Ncm]	Min Value [Ncm]	Mean Value [Ncm]	Min Value [Ncm]
Right posterior	13.1	12	27	25.1
Right anterior	13.2	12	28.3	26.2
Left anterior	15.5	15.1	27.1	25.7
Left posterior	14.1	13.4	26.6	22.8

## Data Availability

The original contributions presented in the study are included in the article, further inquiries can be directed to the corresponding author.
